# Therapeutics and the Lesser Circulation

**Published:** 1894-12-29

**Authors:** Benjamin Ward Richardson


### Therapeutics and the Lesser Circulation.

By Sir Benjamin Ward Richardson, M.D., F.R.S.

II.

In the last paper, on the above subject, an effort was made to indicate the natural balance that exists between the circulating and the aërating mechanisms in the pulmonic structure. The grand question that comes before us therapeutically, after such considerations, is whether we possess in our medical armamentarium any substances which will specifically act upon the circulatory function of the lungs on the one hand, on the aërating function on the other, or on both at the same time. We have also to consider whether there are conditions to which a patient may be subjected, and which specially affect the respiratory system for good or for evil. Let me consider in the first place the action of some medicinal substances.

I observed long ago, in making dissections of the bodies of animals which had died from amyl nitrite, that the condition of the lungs varied much, that sometimes the lungs were of milky whiteness, sometimes of leaden hue, and again of deep, dark red hue. It occurred to me at last that these differences were not accidental, but that they depended upon the mode in which the agent destroyed the life of the animal. Thereupon I made direct inquiry into this subject, and was led to discover that I could, practically, modify the circulation of blood, passing over the lung from the right to the left heart, as I pleased; in other words, I learned that the vessels of the lungs seemed to be influenced by the nitrite in the same manner as the vessels of the skin.

The observation thus stated led me naturally a step further. I inquired as to results of different temporary influences that might be inflicted on the pulmonary organs by the nitrite, and what extremity of influence could be recovered from under conditions favourable to recovery. The results of the labour were most instructive. I found, for instance, that there are four distinct conditions of lung producible by nitrite of amyl.

(1) If a warm blood animal be destroyed by an overwhelming dose of the agent, so that it dies instantly, as it might die from syncope, the lungs are left absolutely bloodless and of pure whiteness. The right side of the heart is in this case paralysed; but exposed to the air immediately after death it often recovers its power of contraction. In this instance the death is really caused by syncope; the nervous paralysis is extended immediately to the heart, and the right ventricle failing to pour out its blood to the lung, the dea'h is so instantaneous that there is no time left for the production of any organic change.

(2) If the death be comparatively slow, if it be preceded by a short interval of muscular prostration, and if it be attended by failure of the muscles of respiration, then the lungs are left charged with dark, tarry blood, but they contain air and are free of bronchial obstruction. Here the lungs and heart have failed together, and the balance of the pulmonary circulation has been fairly maintained.

(3) If the effect of the nitrite be more definitely prolonged, there is produced intense general congestion of the pulmonary vascular system, a congestion so intense that the lungs, full of blood, dark and heavy, will not float in water. The cause of death in this instance is progressive congestion as if from paralysis of the pulmonary vessels; it is the equivalent of congestion of the lungs from long exposure to cold, or from extreme congestive pneumonia.

(4) If the exhibition of the nitrite be continued to a still further prolonged degree, as if life were going on in what is vulgarly called a bad air, where there is persistent want of ventilation, the congestion of the lung takes place at special parts, particularly at the apex, and the congestion so localised may be continued, by exudation of blood into a bronchial passage, with symptoms of hæmoptysis, and with the secondary symptoms which occur in the hæmoptycal state. But if then the nitrite be withdrawn, and pure air be administered, there will be actual attempt at recovery, with absorption of the blood, and with purulent appearance at the spot where the sanguineous exudation took place. In brief, there will be produced certain distinct conditions similar to those seen in pulmonary phthisis at a somewhat advanced stage.

I have traced and followed the changes above described with the utmost care, and have been able to make drawings illustrative of all the different stages quoted. I have gone further even than this; I have noticed the analogous conditions from another substance than the amyl nitrite. The same changes occur under the influence of ethylic alcohol, and up to the fourth stage named they may occur in those who are accustomed to partake freely of alcoholic drinks. The first stage as it is induced by alcohol is partial and short; but the second and third stages are well marked, and in some instances, after prolonged indulgence, I mean indulgence for many years, the last stage may be developed, giving rise to the disease which I have named alcoholic phthsis.

I might dwell upon the condition produced in the lung after the administration of many other chemical or medicinal substances, such as hydrocyanic acid, digitalis, opium, belladonna; but observation is here too scattered to be reliable, for as yet the subject we have in hand is in its earliest infancy, neither physiological nor pathological observation having been systematised in relation to it. The state of the lungs, it is true, is often noticed and recorded; but the record, as a rule, refers simply to the state at the moment observed, without any signification as to the cause which has led to it. I, for my part, know only of direct observation of effect from cause in the two special instances recorded.

It is sufficient at this moment to ask how, when we see such direct effects from cause, how is the effect brought about? What is the reason for it; and, above all, is it due to the immediate action of the agent administered upon the vascular or nervous system of the lungs themselves, or is it dependent purely upon the influence of the heart acting on simple passive structures, indicating only cardiac pressure or cardiac feebleness? Until we can settle this vital question we are indeed at sea.

